# Epigenome-wide association study of diabetic chronic kidney disease progression in the Korean population: the KNOW-CKD study

**DOI:** 10.1038/s41598-023-35485-x

**Published:** 2023-05-20

**Authors:** Hye Youn Sung, Sangjun Lee, Miyeun Han, Woo Ju An, Hyunjin Ryu, Eunjeong Kang, Yong Seek Park, Seung Eun Lee, Curie Ahn, Kook-Hwan Oh, Sue K. Park, Jung-Hyuck Ahn

**Affiliations:** 1 Department of Biochemistry, Ewha Womans University College of Medicine, 25 Magokdong‑ro 2‑gil, Gangseo‑gu, Seoul, 07804 South Korea; 2 Department of Preventive Medicine, Seoul National University College of Medicine, 103, Daehak-ro, Jongro-gu, Seoul, 03080 South Korea; 3 Cancer Research Institute, Seoul National University, Seoul, South Korea; 4 Department of Biomedical Sciences, Seoul National University Graduate School, Seoul, South Korea; 5 Department of Internal Medicine, National Medical Center, Seoul, South Korea; 6 Integrated Major in Innovative Medical Science, Seoul National University College of Medicine, Seoul, South Korea; 7 Department of Internal Medicine, Seoul National University Hospital, 103, Daehak-ro, Jongro-gu, Seoul, 03080 Republic of Korea; 8 Department of Internal Medicine, Ewha Womans University Seoul Hospital, Ewha Womans University College of Medicine, Seoul, South Korea; 9 Department of Microbiology, School of Medicine, Kyung Hee University, Seoul, South Korea

**Keywords:** Genetics, Nephrology

## Abstract

Since the etiology of diabetic chronic kidney disease (CKD) is multifactorial, studies on DNA methylation for kidney function deterioration have rarely been performed despite the need for an epigenetic approach. Therefore, this study aimed to identify epigenetic markers associated with CKD progression based on the decline in the estimated glomerular filtration rate in diabetic CKD in Korea. An epigenome-wide association study was performed using whole blood samples from 180 CKD recruited from the KNOW-CKD cohort. Pyrosequencing was also performed on 133 CKD participants as an external replication analysis. Functional analyses, including the analysis of disease-gene networks, reactome pathways, and protein–protein interaction networks, were conducted to identify the biological mechanisms of CpG sites. A phenome-wide association study was performed to determine the associations between CpG sites and other phenotypes. Two epigenetic markers, cg10297223 on *AGTR1* and cg02990553 on *KRT28* indicated a potential association with diabetic CKD progression. Based on the functional analyses, other phenotypes (blood pressure and cardiac arrhythmia for *AGTR1*) and biological pathways (keratinization and cornified envelope for *KRT28*) related to CKD were also identified. This study suggests a potential association between the cg10297223 and cg02990553 and the progression of diabetic CKD in Koreans. Nevertheless, further validation is needed through additional studies.

## Introduction

Diabetic chronic kidney disease (CKD) is a common cause of end-stage kidney disease. In Korea, almost 50% of end-stage kidney disease (ESKD) cases are caused by diabetic CKD^1^. Diabetic CKD has a poor prognosis, showing increased mortality and rapid progression to ESKD compared to non-diabetic CKD^2,3^.

The etiology of diabetic CKD is multifactorial, including both genetic and environmental factors. High blood glucose levels and blood pressure, prolonged activation of the renin–angiotensin–aldosterone system, and obesity are risk factors associated with the progression of diabetic CKD. However, most of the variability remains unaccounted for by conventional risk factors^4^. For instance, many patients with diabetic CKD with poor glycemic control do not develop renal complications. This discrepancy can be attributed to genetic or epigenetic factors. Genetic codes explain only a fraction of diabetic CKD development, and epigenetic programming, remodeling, and post-translational modifications, such as advanced glycation end products, have been regarded recently as possible physiological mechanisms^5^.

Epigenetic changes, including DNA methylation, histone modification, and miRNA regulation, play major roles in the pathogenesis of diabetic CKD. DNA methylation directly impacts human genome function and serves as a critical regulatory function^6^. Epigenetic changes are thought to be important in determining a predisposition to diabetic CKD. In addition to elevated glucose levels, reactive oxygen species, hypoxia, inflammation, cytokines, drugs, nutrition, and physical activity can also modify epigenetic profiles^5^. Several studies have investigated the DNA methylation profile in diabetic CKD^7–9^. However, most studies were case–control studies, and DNA methylation profiles of renal function decline in diabetic CKD are scarce.

The Korean Cohort Study for Outcomes in Patients with Chronic Kidney Disease (KNOW-CKD) is the largest CKD cohort in Korea to establish the clinical course, risk factors, and adverse outcomes of CKD. More than 50 articles have been published on clinical markers, such as anemia, mineral bone disease, quality of life, and serum biomarkers, such as FGF23, adiponectin, and hepcidin, of CKD in the Korean population using the KNOW-CKD cohort^10^. However, epigenetic biomarkers for the decline in the estimated glomerular filtration rate (eGFR) have not been assessed in the KNOW-CKD cohort. In this study, we aimed to identify epigenetic biomarkers associated with the rapid decline in the eGFR observed in KNOW-CKD subjects using blood samples of diabetic CKD.

## Material and methods

### Ethics and inclusion statement

We acquired written informed consent and blood samples from all participants, and the study was approved by the Institutional Review Boards of Seoul National University Hospital (H-1805-168-948). We also confirmed that all methods were carried out in accordance with relevant guidelines and regulations.

### Data source and study population

The KNOW-CKD cohort was used to perform an EWAS for diabetic CKD progression. The KNOW-CKD study is a prospective multicenter cohort involving 2238 participants with specific causes of chronic kidney disease (CKD) grouped into glomerulonephritis (GN) (n = 810), diabetic nephropathy (DN) (n = 519), hypertensive nephropathy (HTN) (n = 409), polycystic kidney disease (PKD) (n = 364), and unclassified (n = 136) (Supplementary Fig. 1)^10^. Subgroups were defined by pathologic diagnosis, where available, otherwise by clinical diagnosis^11^. GN was identified by the presence of glomerular hematuria or albuminuria, with or without an underlying systemic disease. DN was diagnosed based on albuminuria in individuals with type 2 diabetes mellitus and diabetic retinopathy. HTN was determined by hypertension history and absence of systemic illness associated with renal damage. PKD was diagnosed using unified ultrasound criteria. Other causative diseases were classified as 'unclassified.' The KNOW-CKD cohort has been described in detail elsewhere^11^.

The population was divided into non-progression and progression, based on the eGFR slope of − 2.6 ml/min/1.73 m^2^/year, which was calculated as the median the eGFR slope in the DN from the KNOW-CKD cohort. In addition, the threshold of eGFR slope for HTN was − 2.1 ml/min/1.73 m^2^/year, while it was − 2.6 ml/min/1.73 m^2^/year for DN in a previous study^12^.

This study was a follow-up to the genome-wide association study (GWAS) of the KNOW-CKD group^13^. Out of 519 participants with DN from KNOW-CKD, 434 individuals passed the quality control (QC) for GWAS (Supplementary Fig. 1).

We estimated the appropriate number of participants to be included in the EWAS based on power calculation^14^. Statistical power was estimated from a minimum of 20 to a maximum of 200 participants, assuming a 1:1 ratio of progression to non-progression, 780,000 total CpG sites, 800 targeted CpG sites, minimum detection of |∆ M-value|= 0.01, limma method, FDR threshold of 0.05, and 100 simulations (Supplementary Fig. 2).

Based on power calculation, we attempted to perform the EWAS on 200 participants matched by sex, age, and baseline eGFR between progression and non-progression groups. However, 20 out of 200 participants failed the final epigenomic sample QC process. Therefore, 180 participants (progression: 93, non-progression: 87) were included in the EWAS.

We also performed the pyrosequencing analysis as validation. A total of 78 individuals from DN in KNOW-CKD, excluding those in EWAS, matched by age, sex, and baseline eGFR who passed the QC of pyrosequencing (Supplementary Fig. 1; Supplementary Table 1) were selected. In addition, 55 individuals (41 progression, 14 non-progression) diagnosed with DN from biopsy in the Seoul National University Hospital (SNUH) Human Biobank were included for pyrosequencing analysis (Supplementary Fig. 1). Finally, pyrosequencing analysis was performed based on a total of 133 participants (Supplementary Fig. 1).

### Outcome measurement

The eGFR was calculated using the four-variable Chronic Kidney Disease Epidemiology Collaboration equation^15^. The eGFR slope in the KNOW-CKD cohort was calculated based on linear mixed models with random intercepts using MIXED procedures in SAS software (SAS Institute, Inc., Cary, North Carolina)^16^. The eGFR slope was estimated using creatinine values, measured at every time point; the initiation of the cohort, 6 months after the initiation of the cohort, and at least one follow-up since 2011, every 1–7 years. Only participants that had had eGFR measured at least three times were included. The LMM was fitted, where follow-up time was the dependent variable, and eGFR was the independent variable.

The fixed effect was the effect of “time” on eGFR. The fixed effect represents the average change in eGFR over time in all participants. The random effects are the participant-specific intercepts and slopes of the association between “time” and eGFR. The random intercept represents the variation among the participants in their baseline level of eGFR. The random slope term for "time" captures the variation among the participants in their rate of change of eGFR over time. Therefore, LMM was fitted, allowing each participant to have their own baseline level of eGFR and rate of change in eGFR over time^13,17^.

$${eGFR}_{ij}={\beta }_{0}+{\beta }_{1}{time}_{ij}+{u0}_{i}+{u1}_{i}{time}_{ij}+{\varepsilon }_{ij}$$

*eGFR*_*ij*_: eGFR slope of the i-th subject at the j-th observation time point, *time*_*ij*_: follow-up time of the i-th subject at the j-th observation time point, *β*_*0*_: fixed effect intercept, *β*_*1*_: fixed effect intercept (eGFR slope), *u0*_*i*_: random effect intercept of the i-th subject, *u1*_*i*_: random effect slope of the i-th subject, *ε*_*ij*_: error term (residual).

Hypertension was defined as a systolic blood pressure of ≥ 140 mmHg, diastolic blood pressure of ≥ 90 mmHg, or past medical history. Diabetes mellitus was defined as serum hemoglobin A1C ≥ 6.5%, fasting blood glucose ≥ 126 mg/dl, or a past medical history. CKD progression was defined as an eGFR slope <  − 2.6 mL/min/1.73 m^2^/year.

### Epigenome-wide DNA methylation profiling

Genomic DNA was extracted from leukocytes in the peripheral blood of all samples. Comparison of methylation profiles among the primary outcomes (CKD progression vs. non-progression) was performed using the Illumina Infinium MethylationEPIC platform. The microarray-based DNA methylation levels for individuals were profiled using the Illumina Infinium MethylationEPIC BeadChip kits, which features > 850,000 cytosine-phosphate-guanine (CpG) sites in enhancer regions, gene bodies, promoters, and CpG islands. The DNA methylation array was imaged using a standard Illumina procedure with an Illumina iScan scanner (Illumina, Inc., San Diego, CA, USA).

### Quality control and EWAS

We performed quality control of DNA methylation data extracted from raw intensity data (IDAT), including the signal intensities for each of the probes on the chip with over 1 million probes. To minimize the unintended variation within and between samples, we implemented quantile normalization, which considers the methylated and unmethylated signal intensities separately. We excluded probes with a detection P-value of > 0.05, which can be considered a low-quality signal from all samples. The detection P-value was calculated using the “m + u” method, which compares the total DNA signal (methylated + unmethylated) at each site to the background signal level which is estimated using negative control sites, assuming a normal distribution^18^. CpG sites that failed in one or more samples are filtered based on the detection P-value. We removed the probes on the X or Y chromosome, in addition to the probes affected by single nucleotide polymorphisms (SNPs) without the specification of a certain minor allele frequency^19^. In addition, non-specific binding probes that mapped to multiple locations on the genome were filtered^20^. The annotation was performed by an Illumina Infinium MethylationEPIC BeadChip (EPIC chip), which is a microarray platform designed to DNA methylation across over 860,000 CpG sites in human genome. Finally, 784,864 out of 1,051,815 probes passed the quality control and were included in the EWAS (Supplementary Fig. 3).

CpG sites associated with CKD progression were identified using linear regression models implemented in the limma package in R with an empirical Bayesian framework^21^. The methylation levels at each CpG probe are represented as M-values. The beta-value is a commonly used measure of DNA methylation that ranges from 0 to 1, representing the proportion of methylation at a given CpG site^22^. Conversely, the M-value, or logit-transformed beta-value, is the log2 ratio of the intensities of methylated versus unmethylated probes, calculated as^22^:

$${M}_{i}={log}_{2}\left(\frac{\mathrm{max}({y}_{i,methyl},0+\alpha }{\mathrm{max}\left({y}_{i,methyl}, 0\right)+\alpha }\right)$$

The M-value has the advantage of being symmetrical around zero, and it is often used in statistical analyses, as it allows for more accurate measurement of differential methylation between groups. The M-value ranges from − ∞ to ∞, with values close to zero indicating low methylation and increasingly negative or positive values indicating higher levels of hypomethylation or hypermethylation, respectively^22^.

Since the differences in the various cell types of the whole blood between progression and non-progression can lead to false differentiated methylation regions, the effects of cell proportion on the results of EWAS should be considered^23^. Therefore, in addition to the original model without adjustment for the blood cell proportions (referred to as Model 1), we have also adjusted for blood cell proportions, including T lymphocytes, B cells, monocytes, NK cells, and neutrophils, to remove the false CpG sites from the differences of cell proportions based on the Houseman method (referred to as Model 2). Furthermore, we adjusted for body mass index (BMI) and smoking status (yes/no) as covariates (referred to as Model 3). All summary statistics are provided in Supplementary Table 2.

All results in this study are methylation differences in the primary outcome, diabetic CKD progression versus non-progression. Following the implementation of an epigenome-wide significance threshold of < 0.05 using the false discovery rate (FDR)^24^, the number of CpG sites were reduced from a total of 784,864 to 9,809, 8,900, and 8,690 in Model 1, Model 2, and Model 3, respectively (Supplementary Fig. 3; Supplementary Fig. 4).

Subsequently, CpG sites without an annotation for gene symbols were removed (7252, 6537, and 6328 CpG sites in the Model 1, Model 2, and Model 3, respectively). We only selected CpG sites located in the promoter regions (promoters were defined as regions located between 0 and 1500 bp upstream of transcriptional start sites (TSS), 5′UTR, and the 1st exon). There were 3837, 3462, and 3261 CpG sites in the Model 1, Model 2, and Model 3, respectively. In addition, CpG sites located in the shelf and shore regions of the CpG island (CGI) were selected (843, 774, and 742 CpG sites in the Model 1, Model 2, and Model 3, respectively). Furthermore, CpG sites with a more restricted FDR threshold (FDR < 0.005) were selected to perform pyrosequencing and in-silico functional analysis (197, 157, and 157 CpG sites in the Model 1, Model 2, and Model 3, respectively).

We used the top five percentile |∆ M-value| as the threshold in the distribution of |∆ M-value| to exclude false positive CpG sites. Since |∆ M-value| has a left skewed distribution, we determined the top five percentile values based on a non-parametric bootstrapping resampling method (alpha = 0.05, the number of resampling = 1000) (Supplementary Fig. 5). The top five percentile values of the quantile were estimated (quantile [95% CI] = 0.2954 [0.2915, 0.3002]) based on the resampling distribution. In addition, the top five percentile values from the EWAS with the adjustment for blood cell proportions were estimated as quantile [95% CI] = 0.3007 [0.2958, 0.3045] (Supplementary Fig. 5). Furthermore, the top five percentile values from the EWAS with the adjustment for not only blood cell proportions but also BMI and smoking status were estimated as quantile [95% CI] = 0.0382 [0.0371, 0.0393] (Supplementary Fig. 5). Therefore, we selected highly significant CpG sites with an │∆M-value│ ≥ 0.30 in Model 1 and 2 (17 and 15 CpG sites in Model 1 and Model 2), with an │∆M-value│ ≥ 0.038 in Model 3 (12 CpG sites in Model 3), respectively.

Quality control and genome-wide analyses were conducted using the Minfi package from the Bioconductor platform in R^25,26^.

### Pyrosequencing: replication analysis

The 17 candidate CpG sites used in pyrosequencing analysis were selected from the results of EWAS with no adjustment for blood cell proportions (Supplementary Fig. 3; Supplementary Fig. 6). The pyrosequencing primer was designed using the PyroMark Assay Design SW 2.0 software (QIAGEN) under the following three conditions: (1) maximum amplicon length < 200 bp, (2) primer set score ≥ 75, and (3) primers attached to CpG sites were excluded (Supplementary  Fig. 6). Ultimately, only 11 out of 17 CpG sites (cg11513352, cg10297223, cg22773662, cg03503634, cg04089320, cg20746451, cg14279121, cg15280188, cg11508872, cg21285133, and cg02990553 within genes *DOC2A*, *AGTR1* (Angiotensin II receptor type 1), *MIEF1*, *TRAF6*, *EMB*, *SMARCAD1*, *OSBPL9*, *ASPSCR1*, *RAB14*, *ANP32E*, and *KRT28* (Keratin 28), respectively) were available to undergo pyrosequencing analysis under these three conditions. The primer sequences used in this study are listed in Supplementary Table 3.

For each assay, bisulfite-converted DNA was amplified using PCR, using the instructions provided by the manufacture of by the PyroMArk PCR kit (QIAGEN). The PCR product was bound to magnetic streptavidin beads. Quality control of the pyrosequencing data was performed using the PyroMark Q48 software. All samples passed the quality control process. Sequencing was performed on a PyroMark Q48 Autoprep system using the PyroMark Q48 Advanced CpG Reagents (QIAGEN) according to the manufacturer’s instructions.

The percentage of DNA methylation at specific CpG sites was estimated using the PyroMark Q48 Autoprep 2.4.2 software (QIAGEN) and exported to the R statistical environment. Subsequently, linear regressions were performed for each CpG site covered by the assay, as well as for the average methylation value across the region.

We performed a linear regression analysis with the average methylation level of methylated cytosines as the dependent variable and progression/non-progression as the independent variable to select CpG sites that show differential methylation levels between these two groups (progression/non-progression)^27^. The beta estimation in the regression was used to calculate the difference in methylation levels between the two groups (Supplementary Table 3).

### Phenome-wide association study

We performed the PheWAS for cg10297223 and cg02990553 CpG sites based on the variables (phenotypes) in KNOW-CKD cohort. Based on a total of 1,028 variables in KNOW-CKD cohort, we excluded 719 variables with a missing rate over 10%. Of the remaining 309 variables, including in the PheWAS, 144 were continuous and 165 were categorical variables. The association between each |M-value| of the CpG sites and the phenotype was estimated using linear or logistic regression models according to the continuous or categorical phenotypes, respectively. The statistical significance threshold for PheWAS was also set at FDR < 0.05 using the Benjamini–Hochberg method^24^.

### In silico functional analysis

We further performed functional annotation analysis, such as the analysis of disease-gene network (DGN), reactome (RA) pathways, and protein–protein interaction (PPI) network, to identify the biological mechanisms of CpG sites. DNG has been used to identify cross-phenotypes associated with selected genes from CpG sites using DisGeNET^28^. We also used the RA database to annotate gene sets for biological pathways^29^. The PPI network was constructed using the Search Tool for the Retrieval of Interacting Genes (STRING; http://string.embl.de/) with a confidence score ≥ 0.99 to identify the functional interactions between proteins^30^. Statistical significance was determined by a false discovery rate (FDR)-corrected P-value of < 0.05. Furthermore, we identified the Expression Quantitative Trait Methylation (eQTM) based on a human whole-blood epigenome-wide association study from the Human Kidney eQTM by Susztak Lab (available on https://susztaklab.com/Kidney_meQTL/index.php)^31^. Network illustrations from the functional analyses were constructed using the Cytoscape software (version 3.9.1) via Rcy3^32,33^.

### DNA methyltransferase (DNMT) inhibitor treatment

HEK 293 cells were cultured in Dulbecco’s modified Eagle’s medium (Thermo Fisher Scientific, Waltham, MA, USA) supplemented with 10% fetal bovine serum (FBS, Thermo Fisher Scientific), 100 U/ml penicillin (Thermo Fisher Scientific), and 100 μg/ml streptomycin (Thermo Fisher Scientific) in an atmosphere containing 95% humidified air and 5% CO_2_ at 37 °C. To demethylate methylated CpG sites, HEK 293 cells were treated with increasing concentrations (0, 5, 10, and 20 μM) of 5-aza-2′-deoxycytidine (Sigma-Aldrich, St. Louis, MO, USA) for 72 h, which was replaced daily. Inhibition of methylation was examined by pyrosequencing analysis, and changes in *AGTR1* expression were measured by reverse-transcription quantitative polymerase chain reaction (RT-qPCR).

### RNA preparation and reverse-transcription quantitative polymerase chain reaction (RT-qPCR)

Total RNA was extracted from HEK 293 cells using the RNeasy Mini Kit (Qiagen, Valencia, CA, USA), according to the manufacturer’s protocol. One microgram of total RNA was converted to cDNA using Superscript II reverse transcriptase (Invitrogen, Carlsbad, CA, USA) and oligo-(dT)12–18 primers (both from Thermo Fisher Scientific) according to the manufacturer’s instructions. qRT-PCR was performed in a 20 μl reaction mixture containing 1 μl cDNA, 10 μl SYBR Premix EX Taq (Takara Bio, Otsu, Japan), 0.4 μl Rox reference dye (50×, Takara Bio), and 200 nM primers for each gene. The following primer sequences were used in this study:

- *AGTR1* (forward), 5′-GCCCTTTGGCAATTACCTATGT-3′;
- *AGTR1* (reverse), 5′-CGTGAGTAGAAACACACTAGCGT-3′;
- *GAPDH* (forward), 5′-AATCCCATCACCATCTTCCA-3′;
- *GAPDH* (reverse), 5′-TGGACTCCACGACGT ACTCA-3′.

The reactions were run on a 7500 Fast Real-Time PCR System (Applied Biosystems, Foster City, CA, USA) at 95 °C for 30 s, followed by 40 cycles at 95 °C for 3 s and 60 °C for 30 s, and a single cycle at 95 °C for 15 s, 60 °C for 60 s, and 95 °C for 15 s to generate dissociation curves. All PCR reactions were performed in triplicate, and the specificity of the reaction was determined by melting curve analysis. Comparative quantification of each target gene was performed based on the cycle threshold (Ct) normalized to *GAPDH*, using the ΔΔCt method^34^.

## Results

### General characteristics of study population

Table 1 shows the clinical and demographic characteristics of 180 diabetic participants with CKD (93 progression versus 87 non-progression) based on the KNOW-CKD cohort study. The mean age was 59.1 years, men accounted for 65% of the participants, and most participants had hypertension (98.3%). Urine albumin, urine protein, UACR, UPCR, 24-h urine protein, and 24-h urine phosphorus levels were higher in the progression group than in the non-progression group (Table 1).

**Table 1:** General characteristics of diabetic chronic kidney disease based on the KoreaN cohort study for Outcome in patients With Chronic Kidney Disease (KNOW-CKD) cohort study.

	Total (N = 180)	Progression (N = 93)	Non-progression (N = 87)	P-value
	Mean (SD)	Mean (SD)	Mean (SD)	
Age at baseline	59.1 (8.0)	58.6 (8.4)	59.6 (7.5)	0.43
Systolic BP (mmHg)	131.4 (15.0)	131.9 (15.3)	130.9 (14.7)	0.68
Diastolic BP (mmHg)	74.5 (9.7)	74.1 (9.5)	74.9 (9.9)	0.58
Body mass index (kg/m^2^)	25.0 (3.0)	25.1 (2.7)	25.0 (3.4)	0.73
White blood cells (/mm^3^)	7124.0 (2067.6)	7036.3 (2121.8)	7217.7 (2016.0)	0.56
Hemoglobin (g/dL)	11.8 (1.7)	11.7 (1.7)	11.9 (1.6)	0.37
Urine albumin (mg/dL)	1169.3 (1444.0)	1803.0 (1688.4)	491.9 (626.1)	< 0.01
Urine protein (mg/dL)	167.7 (207.4)	257.0 (245.1)	72.3 (86.1)	< 0.01
UACR	1.3 (1.6)	2.0 (1.8)	0.5 (0.8)	< 0.01
UPCR	1.9 (2.4)	2.9 (2.7)	0.8 (1.1)	< 0.01
24-h urine protein (g)	1.9 (2.2)	3.0 (2.6)	0.8 (0.8)	< 0.01
24-h urine phosphorus (g)	0.9 (1.5)	1.2 (2.0)	0.5 (0.2)	< 0.01
eGFR (ml/min/1.73 m^2^)	37.4 (13.2)	37.4 (13.0)	37.4 (13.5)	0.98
eGFR slope (ml/min/1.73 m^2^/year)	− 3.0 (2.2)	− 4.7 (1.5)	− 1.2 (1.1)	< 0.01

	Median (IQR)	Median (IQR)	Median (IQR)	P-value
Follow up (years)	3.9 (2.3)	3.3 (1.9)	4.3 (3.0)	< 0.001

	N (%)	N (%)	N (%)	P-value
Sex (male)	117 (65.0)	62 (66.7)	55 (63.2)	0.62
Hypertension	177 (98.3)	93 (100.0)	84 (96.6)	0.11

BP, blood pressure; eGFR, estimated glomerular filtration rate; IQR, interquartile Rang.

Furthermore, Supplementary Table 1 shows the general characteristics of 78 DN from the KNOW-CKD cohort and 55 from the biopsy in SNUH Human Biobank for pyrosequencing analysis. Diastolic blood pressure (BP), Urine albumin, urine protein, UACR, UPCR, and 24-h urine protein levels were higher in the progression group than in the non-progression group among the 78 participants. However, only a limited number of variables were investigated among the 55 participants.

### Differentially methylated CpG sites

**Figure 1:**
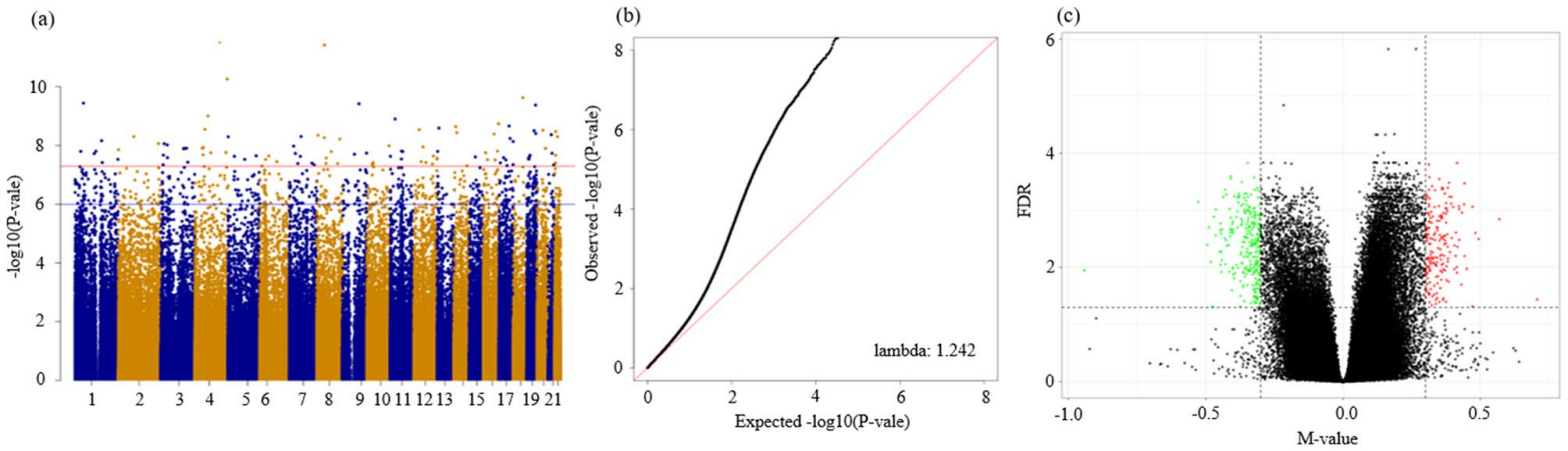
Visualization of methylated probes. (**a**) Manhattan and (**b**) quantile–quantile plots of the epigenome-wide association study for CKD progression. (**c**) Volcano plot showing differentially methylated CpG sites. The x-axis presents the M-value of the difference in signal intensity between the primary outcome for each probe. The y-axis represents the -log10 (P-value). Significant CpG sites (FDR < 0.05 and |∆ M-value|> 0.30) are highlighted in red and green. CpG sites highlighted as red and blue are those that were hypermethylated and hypomethylated compared to the non-progression, respectively. FDR, false discovery rate.

In the results with no adjustment for blood cell proportion, 17 CpG sites remained based on the FDR < 0.005 and |∆ M-value|≥ 0.3 threshold (Fig. 1; Supplementary Fig. 3). According to the results of the adjustment for blood cell proportion with the same threshold, 15 CpG sites were identified (Supplementary Fig. 3). Based on the results of the adjustment for blood cell proportion, BMI, and smoking status, 12 CpG sites remaining based on the FDR < 0.005 and |∆ M-value|≥ 0.038 threshold (Supplementary Fig. 3). Of the 15 CpG sites, only 14 CpG sites (cg20746451, cg01490296, cg10297223, cg02990553, cg06205244, cg21285133, cg04089320, cg22773662, cg03503634, cg15280188, cg21285782, cg14279121, cg11508872, cg26296769) were identical and of the 12 CpG sites, only seven CpG sites (cg20746451, cg01490296, cg10297223, cg02990553, cg06205244, cg21285133, cg04089320) were identical to the original 17 CpG sites, respectively (Table 2; Supplementary Fig. 3).

**Table 2:** Candidate CpG sites for primer design for the external validation based on pyrosequencing analysis.

CpGs	Chr	Position	Gene	Feature	cgi	ΔM-value	P-value	FDR
No adjustment for blood cell proportions (17 CpG sites)								
cg02990553^a^	17	38955770	*KRT28*	1stExon	Shelf	0.350	3.10E−08	2.84E−04
cg21285133^a^	1	150209464	*ANP32E*	TSS1500	Shore	0.305	1.68E−07	5.30E−04
cg11508872^a^	9	123964527	*RAB14*	TSS200	Shore	0.313	4.93E−07	7.81E−04
cg01490296	10	13205619	*MCM10*	5′UTR	Shore	0.366	1.04E−06	1.10E−03
cg15280188^a^	17	79934854	*ASPSCR1*	TSS1500	Shore	− 0.343	3.03E−06	1.91E−03
cg14279121^a^	1	52194898	*OSBPL9*	TSS1500	Shore	− 0.325	3.22E−06	1.96E−03
cg04089320^a^	5	49737641	*EMB*	TSS1500	Shore	− 0.384	3.37E−06	2.00E−03
cg03503634^a^	11	36531973	*TRAF6*	TSS200	Shore	− 0.463	6.03E−06	2.74E−03
cg26296769	6	43025862	*KLC4*	TSS1500	Shore	0.304	7.43E−06	3.08E−03
cg10297223^a^	3	148414649	*AGTR1*	TSS1500	Shore	0.365	7.86E−06	3.18E−03
cg20746451^a^	4	95127703	*SMARCAD1*	TSS1500	Shore	− 0.426	8.61E−06	3.35E−03
cg22773662^a^	22	39899355	*MIEF1*	5′UTR	Shore	0.352	8.75E−06	3.37E−03
cg06205244	6	86299452	*SNX14*	5′UTR	Shelf	0.306	8.84E−06	3.40E−03
cg23715505	10	115613921	*NHLRC2*	TSS1500	Shore	− 0.324	1.07E−05	3.80E−03
cg24577191	19	18344621	*PDE4C*	5′UTR	Shore	0.318	1.10E−05	3.86E−03
cg11513352^a^	16	30022537	*DOC2A*	5′UTR	Shore	0.406	1.23E−05	4.12E−03
cg21285782	1	154530958	*UBE2Q1*	1stExon	Shore	− 0.330	1.28E−05	4.21E−03
Adjustment for blood cell proportions (15 CpG sites)								
cg14279121	1	52194898	*OSBPL9*	TSS1500	Shore	− 0.324	4.79E−06	2.82E−03
cg21285133	1	150209464	*ANP32E*	TSS1500	Shore	0.309	1.97E−07	7.21E−04
cg21285782	1	154530958	*UBE2Q1*	1stExon	Shore	− 0.338	1.16E−05	4.52E−03
cg10297223	3	148414649	*AGTR1*	TSS1500	Shore	0.368	9.04E−06	3.90E−03
cg20746451	4	95127703	*SMARCAD1*	TSS1500	Shore	− 0.444	5.27E−06	2.94E−03
cg04089320	5	49737641	*EMB*	TSS1500	Shore	− 0.386	3.72E−06	2.53E−03
cg26296769	6	43025862	*KLC4*	TSS1500	Shore	0.302	1.18E−05	4.58E−03
cg06205244	6	86299452	*SNX14*	5′UTR	Shelf	0.309	9.81E−06	4.08E−03
cg11508872	9	123964527	*RAB14*	TSS200	Shore	0.313	6.49E−07	1.04E−03
cg01490296	10	13205619	*MCM10*	5′UTR	Shore	0.371	1.15E−06	1.37E−03
cg03503634	11	36531973	*TRAF6*	TSS200	Shore	− 0.475	4.69E−06	2.80E−03
cg08054244	14	88789549	*KCNK10*	1stExon	Shore	0.444	5.19E−06	2.92E−03
cg02990553	17	38955770	*KRT28*	1stExon	Shelf	0.354	3.49E−08	3.91E−04
cg15280188	17	79934854	*ASPSCR1*	TSS1500	Shore	− 0.345	3.48E−06	2.46E−03
cg22773662	22	39899355	*MIEF1*	5′UTR	Shore	0.361	7.03E−06	3.41E−03
Adjustment for blood cell proportions, BMI, and smoking status (12 CpG sites)								
cg21285133	1	150209464	*ANP32E*	TSS1500	Shore	0.045	1.87E−07	6.42E−04
cg24962873	3	11312541	*ATG7*	TSS1500	Shore	0.039	5.99E−07	9.81E−04
cg10297223	3	148414649	*AGTR1*	TSS1500	Shore	0.060	7.28E−06	3.56E−03
cg07321536	4	39459897	*LIAS*	TSS1500	Shore	− 0.040	3.30E−06	2.38E−03
cg20746451	4	95127703	*SMARCAD1*	TSS1500	Shore	− 0.065	4.10E−06	2.66E−03
cg06205244	6	86299452	*SNX14*	5′UTR	Shelf	0.052	1.06E−05	4.40E−03
cg19933320	7	64125401	*ZNF107*	TSS1500	Shore	0.039	2.69E−06	2.12E−03
cg10551778	8	359909	*FBXO25*	5′UTR	Shelf	0.039	1.78E−07	6.32E−04
cg01490296	10	13205619	*MCM10*	5′UTR	Shore	0.062	1.60E−06	1.61E−03
cg24299813	11	60685836	*TMEM109*	5′UTR	Shelf	0.045	6.00E−07	9.81E−04
cg02990553	17	38955770	*KRT28*	1stExon	Shelf	0.060	4.26E−08	3.97E−04
cg22921692	19	9930722	*FBXL12*	TSS1500	Shore	0.041	2.23E−08	2.92E−04

Chr, chromosome; cgi, CpG island; CpG, Cytosine-phosphate-Guanine; BMI. body mass index; FDR, false positive rate.

^a^Eleven out of 17 CpG sites were available for primer design.

**Figure 2:**
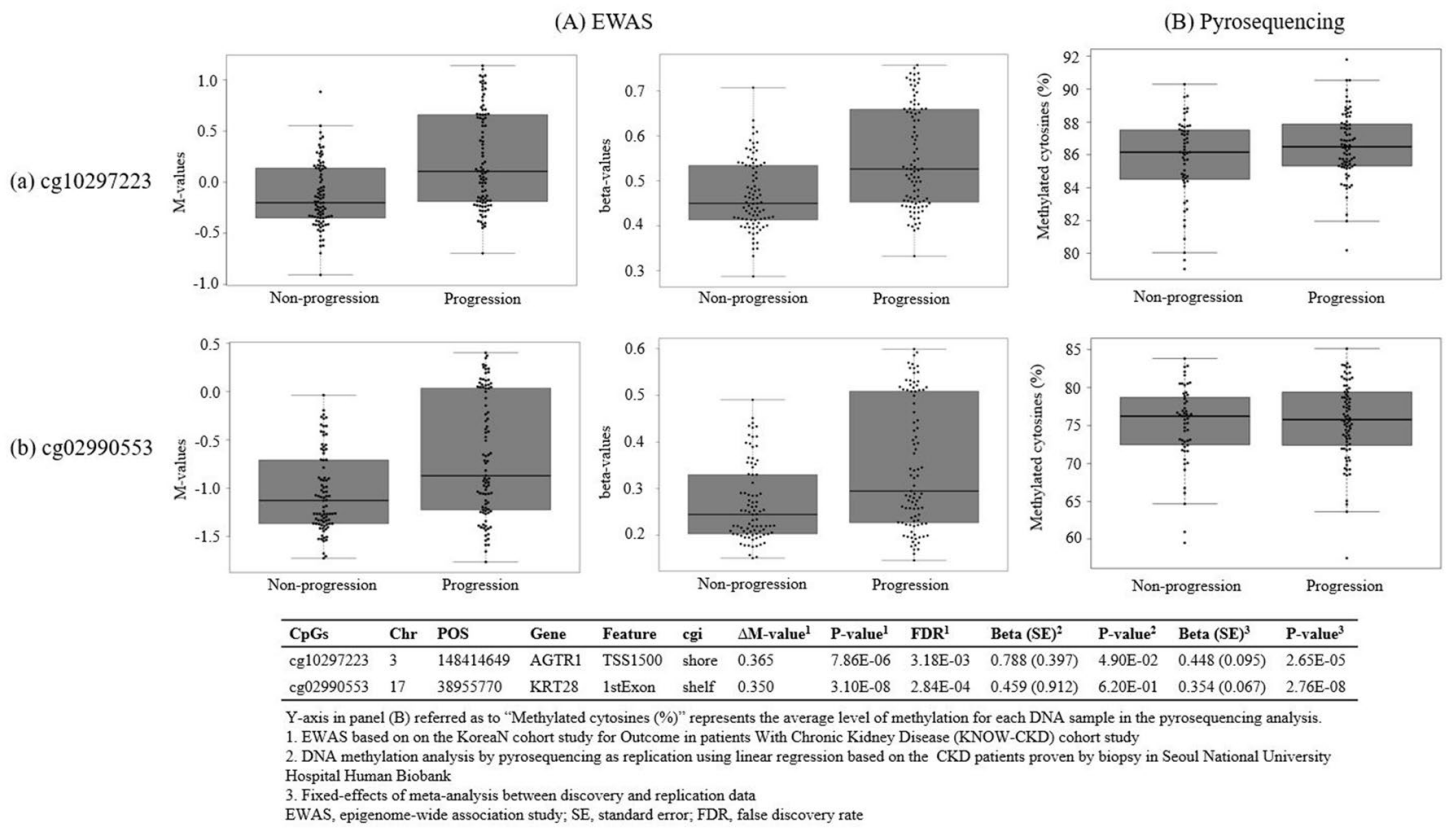
DNA methylation analysis by epigenome-wide association study as discovery and pyrosequencing as validation for the progression of chronic kidney disease (CKD) in diabetic CKD patients. Beeswarm and box plots shows the DNA methylation values of two CpG sites. (**A**) The M-values and beta-values of epigenome-wide association study based on EPIC BeadChip for (a), cg02990553 on *AGTR1* (b), and cg10297223 on *KRT28*. (**B**) The percentage of differentially methylated CpG sites using pyrosequencing were generated for (a), cg02990553 on *AGTR1* (b), and cg10297223 on *KRT28*.

The results of pyrosequencing provided information about the proportion of methylated cytosines in each DNA sample, as well as information about the average level of methylation at individual CpG sites^35^. Of the 11 candidate CpG sites available for pyrosequencing primer design, six genes (*DOC2A*, *MIEF1*, *EMB*, *SMARCAD1*, *ASPSCR1*, and *ANP32E*) could not be considered validated due to the inconsistent direction of effect size with the discovery results (Supplementary Table 3). Out of the remaining five genes, three genes, *TRAF6*, *OSBPL9*, and *RAB14*, were difficult to validate due to the small effect sizes despite the consistent direction of effect size with the discovery results of EWAS. cg10297223 on *AGTR1* (EWAS: ∆M-value = 0.365, FDR = 3.18E−03, pyrosequencing: Beta (SE) = 0.788 (0.397), P-value = 4.90E−02) was considered potentially validated, whereas cg02990553 on *KRT28* (EWAS: ∆M-value = 0.350, FDR = 2.84E−04, pyrosequencing: Beta (SE) = 0.459 (0.912), P-value = 6.10E−01) only demonstrated a consistent direction of effect size without sufficient evidence for statistical validation by pyrosequencing analysis, respectively (Table 2; Fig. 2). In addition, both cg10297223 and cg02990553 were associated with seven phenotypes (24-h urine protein, 24-h urine phosphorus, urine albumin, urine protein, UPCR, UACR, and eGFR slope) based on the PheWAS (Table 3).

**Table 3:** Phenome-wide association study based on M-values of cg10297223 (*AGTR1*) and cg02990553 (*KRT28*).

Phenotypes	Effect size	P-value	FDR
cg10297223 (*AGTR1*)			
24-h urine protein (mg/day)	1566.788	3.726E−07	2.787E−05
24-h urine phosphorus (mg/day)	1070.956	2.299E−07	2.787E−05
Urine albumin	824.337	9.602E−06	2.394E−04
Urine protein	119.347	8.045E−06	2.149E−04
UPCR	1.408	3.809E−06	1.476E−04
UACR	951.300	2.330E−06	1.089E−04
eGFR slope	− 1.015	3.106E−04	6.489E−03
cg02990553 (*KRT28*)			
24-h urine protein (mg/day)	2058.406	2.936E−07	2.787E−05
24-h urine phosphorus (mg/day)	1231.686	3.947E−06	1.476E−04
Urine albumin	1161.956	8.292E−07	5.169E−05
Urine protein	162.965	1.548E−06	8.268E−05
UPCR	1.968	3.355E−07	2.787E−05
UACR	1362.769	8.483E−08	2.787E−05
eGFR slope	− 1.511	2.223E−05	5.224E−04

FDR, false discovery rate; UPCR, urinary protein-to-creatinine ratio; UACR, urinary albumin-to-protein ratio; eGFR, estimated glomerular filtration rate.

### In silico functional analysis

**Figure 3:**
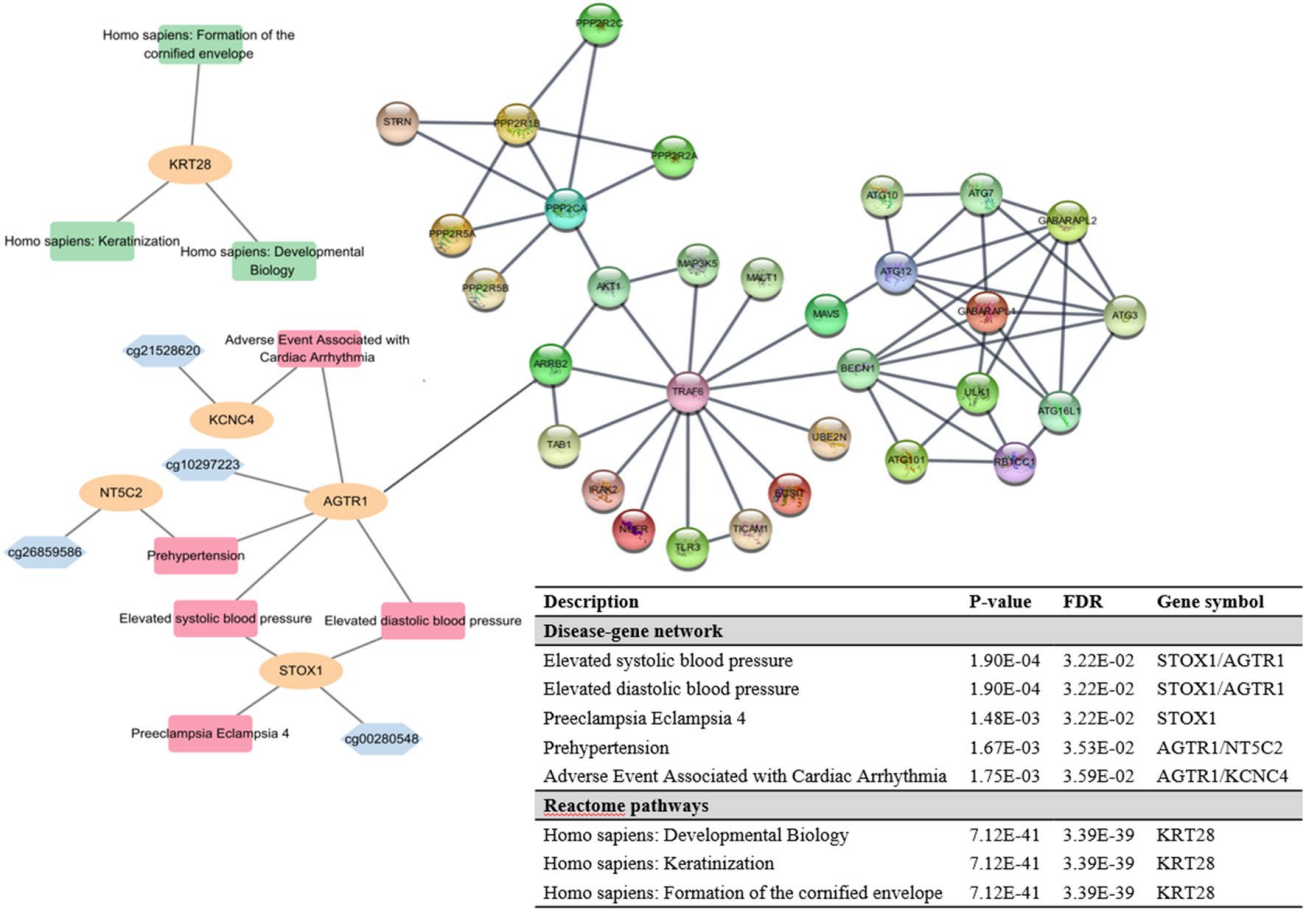
Functional analysis for cg10297223 on *AGTR1* and cg02990553 on *KRT28* which are associated with diabetic CKD. Blue, orange, red, and green nodes indicate CpG sties, gene symbols, disease-gene networks, and Reactome pathways, respectively. Nodes with molecular structure indicate PPI networks. CKD, chronic kidney disease; PPI, protein–protein interaction.

Based on the functional analysis of the DGN, *AGTR1* and *STOX1* were associated with elevated systolic (FDR = 3.22E−02) and diastolic BP (FDR = 3.22E−02) (Fig. 3). In addition, *AGTR1* and *NT5C2* were associated with pre-hypertension (FDR = 3.53E−02), and *AGTR1* and *KCNC4* were associated with adverse events associated with cardiac arrhythmia (FDR = 3.59E−02). *AGTR1* was also associated with *TRAF6* based on the PPI network. *KRT28* was involved in the biological pathways of developmental biology, keratinization, and the formation of the cornified envelope (FDR = 3.39E−39) based on the RA pathways (Fig. 3; Supplementary Table 4). In addition, four CpG sites (g15280188, cg14279121, cg04089320, and cg11513352) among a total of 17 top CpG sites were identified as eQTM (Supplementary Table 5).

### AGTR1 expression was regulated by epigenetic DNA methylation

**Figure 4:**
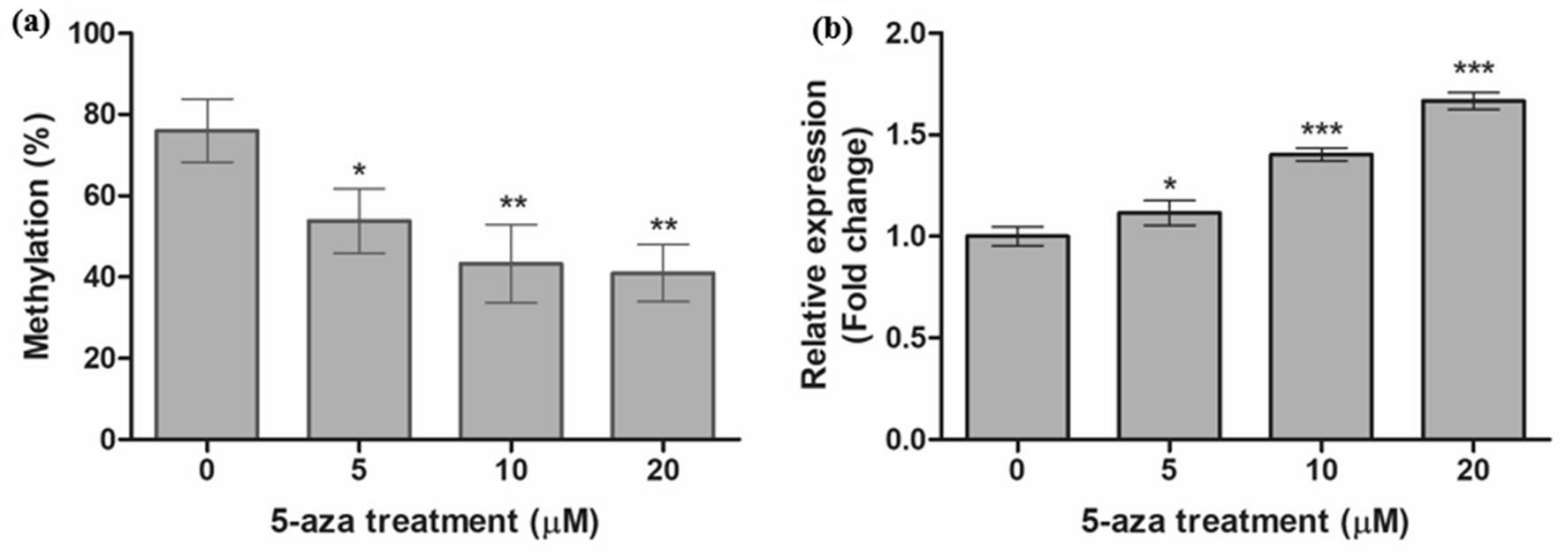
Modulation of *AGTR1* mRNA expression following demethylation in HEK 293 cells. HEK 293 cells were treated for 72 h with various concentration of 5-aza-2′-deoxycytidine. After treatment, demethylation of *AGTR1* promoter CpG site (cg10297223) was confirmed by pyrosequencing analysis (**a**) and the expression of *AGTR1* mRNA was measured by RT-qPCR (**b**). Data are presented as the mean ± SD from three independent experiments. Statistical analyses were performed using one-way ANOVA with Dunnett’s multiple comparison post-test for comparing significance with untreated control (*P < 0.05, **P < 0.01, ***P < 0.001). 5-aza, 5-aza-2′-deoxycytidine.

To determine whether the expression of *AGTR1* mRNA was epigenetically modulated, we treated HEK 293 cells with the DNA methyltransferase inhibitor 5-aza-2′-deoxycytidine. The expression of *AGTR1* mRNA was quantified by RT-qPCR and the methylation status of the CpG site (cg10297223) within *AGTR1* was determined by pyrosequencing analysis. After treatment with 5-aza-2′-deoxycytidine, the expression of *AGTR1* mRNA was significantly restored (~ 1.67-fold) in a dose-dependent manner, which occurred concurrently with the decreased methylation status of the *AGTR1* promoter CpG site (Fig. 4). These results indicate that *AGTR1* expression is regulated by a DNA methylation-dependent mechanism.

## Discussion

In our study, EWAS was performed to select CpG sites that were differentially methylated during the progression of diabetic CKD in the Korean population. External replication analysis was performed using pyrosequencing, focusing on the top-ranked candidate CpG sites. Consequently, cg10297223 on *AGTR1* and cg02990553 on *KRT28* were found to be significant CpG markers, and gene-level functional analysis was performed to confirm that the two CpG sites share biological mechanisms with diabetic CKD progression based on existing knowledge or hypotheses.

The *AGTR1* is a G-protein-coupled transmembrane receptor located at the end of the renin–angiotensin–aldosterone system (RAAS) cascade^36^. The RAAS cascade is a major regulator of systemic arterial blood pressure, fluid, and electrolyte balance^37^, primarily functions in the second stage of the embryo, and plays essential roles in neonates, maintenance of peripheral vascular resistance, and renal blood flow^38^.

In addition, the interaction between *AGTR1* and angiotensin II, which is released from mesangial cells, has been demonstrated to activate the inflammatory cascade by regulating protein kinase C and the mitogen-activated protein kinase (MAPK) pathway^39^ and induce the expression of growth factors and proliferative cytokines to sustain the generation of nephrotoxic reactive oxygen, resulting in inflammation, fibroblast formation, and collagen deposition^40^. Angiotensin II also activates signaling of the NF-κB pathways, which are activated by TNF-receptor-associated factor (TRAF), leading to inflammation^41–43^.

Therefore, it has been suggested that pathogenic mutations leading to the absence or defects in *AGTR1* can induce fatal phenotypes^44^. Moreover, chronic activation of the RAAS is recognized as a critical factor in CKD progression^45,46^. In addition, a non-functioning RAAS cascade results in kidney damage under neonatal or hypoxic conditions^47^.

A study on *AGTR1*-related CKD in RAAS at the GWAS level was also reported in a systematic review and meta-analysis^48^. In the Chronic Renal Insufficiency Cohort study based on Caucasian and African American populations in 2015, the association between RAAS-related genes with CKD was reported, but *AGTR1* was not found to be significantly associated with CKD^49^. However, another GWA study in an African American population reported an association between *AGTR1* and diabetic ESKD^50^. Nevertheless, previous studies have only reported an association between the alleles of SNPs in *AGTR1* and CKD, and studies on gene activation, such as gene expression or methylation of *AGTR1*, especially in the Korean population, have not yet been described.

*KRT28* encodes a member of the type I (acidic) keratin family, which belongs to the superfamily of intermediate filament (IF) proteins^51^. Previous studies have reported that a cornified envelope or keratinization, which is associated with *KRT28*, is also associated with CKD.

The components of the cornified envelope were considerably reduced in participants with CKD compared to that of the control group in a previous study^52^. It has been reported that treatment with emollients can reduce the thickness and density of scales and noticeably improve the quality of life of CKD^53^.

Moreover, acquired perforating dermatosis (APD), which is caused by chronic friction leading to epithelial proliferation, abnormal keratinization, and decreased blood supply due to microangiopathy, is often associated with underlying systemic diseases, such as diabetes mellitus and CKD^54^. APD most often occurs after starting dialysis in CKD^55^. Kidney damage is known to affect wound healing^56^. Research data on rats also showed an exacerbating effect of CKD on wound healing, which is mediated by the disruption of keratinization and delayed granulation^56^. In addition, veiled chronic inflammatory conditions, low rates of angiogenesis, and cell proliferation also contribute to poor wound healing^57^. Although several *KRT* series genes related to keratinization have been reported in previous studies, *KRT28* (particularly as an epigenetic marker in Korean populations) was implicated for the first time in our study^58^.

Previous studies based on similar hypotheses have been reported for populations other than Koreans. The previous study has reported the enhancement of renal regulatory regions and their correlation with gene expression changes, including epidermal growth factor, related to kidney damage and impaired function using methylation probes^59^. Another study reported similar correlation results for individuals receiving kidney transplants or dialysis, demonstrating the ability to analyze transplant recipients alongside individuals receiving dialysis to improve the performance of future EWAS for ESKD^60^.

Our study had several limitations that need to be acknowledged. First, although we selected candidate CpG sites for performing external replication analysis using pyrosequencing, epigenome-wide replication analysis could not be performed owing to the lack of Korean or Asian-based CKD cohorts with epigenomic databases. Nevertheless, since the KNOW-CKD cohort, which forms the basis of the current study, has almost completed the recruitment of an additional 1500 CKD participants for phase II and has started follow-up (https://clinicaltrials.gov/ct2/show/NCT03929900), we will be able to conduct epigenome-wide validation analysis in the future^13^.

Second, because the DNA samples used in our study were derived from peripheral blood samples, there is limited information on the association between whole blood DNA methylation profiles and kidney tissue-specific DNA methylation differentiation, in part due to the heterogeneity of cell types within the kidney. However, a previous study suggested that blood DNA methylation analysis is valuable because it can reflect changes in DNA methylation in the tissues associated with the phenotypes^61^. Nevertheless, the establishment of a biobank of kidney biopsies is needed to improve tissue-specific DNA methylation analysis for kidney disease in the future^61^.

Although we used the threshold of |∆ M-value| as 0.3 and 0.0038 for EWAS with/without adjustment of blood cell proportions and with adjustment of blood cell proportions, BMI, and smoking status, respectively, in order to exclude false positive CpG probes, there is a possibility that CpG probes with a small difference were not considered as candidate CpG sites for the validation due to the high threshold of the |∆ M-value|.

There was a possibility that cg10297223 and cg02990553 could be validated in the pyrosequencing analysis in our study. However, although cg10297223 had a significant raw P-value (P-value < 0.05), both CpG sites had no statistical significance in FDR or Bonferroni correction (Supplementary Table 3). In future, we hope to validate this finding, utilizing the KNOW-CKD phase II cohort which, as already mentioned, will include an additional 1500 CKD participants—the recruitment of these participants is almost completed^13^.

Since the Human Kidney eQTM results gathered by Susztak Lab were sampled from a different ethnic group than the ethnicity of the cohort used in our study^31^, there may be an association between Korean-specific epigenetics markers and gene expression that has not yet been identified.

Furthermore, the use of 5-aza-2′-deoxycytidine results in the demethylation of CpGs throughout the genome of cells, making it challenging to apply this treatment for the causal analysis of effects. Therefore, caution should be exercised when interpreting the results, as the observed changes may not be directly attributable to the targeted CpG sites^62^.

Despite these limitations, our study had several strengths. First, although epigenome-wide replication analysis could not be performed, functional annotation analysis was conducted in silico to elucidate the biological mechanisms of the CpG sites identified in our study. Moreover, based on PheWAS, CpG sites associated with diabetic CKD in our study were confirmed to be appreciably associated with different phenotypes related to CKD progression.

Second, although the DNA samples used in our study were whole blood DNA samples, a gene or that of the same family reported as GWAS-level in previous studies was also identified in our findings. Moreover, our study demonstrated that epigenetic markers affect gene expression and proteomic production more in the central dogma than at the GWAS-level. Furthermore, since our CpG markers were extracted from a clinically accessible peripheral blood sample, they can be used as diagnostic markers in the future.

We have identified two epigenetic markers (cg10297223 on *AGTR1* and cg02990553 on *KRT28*) that show a potential association with diabetic CKD progression in the Korean population. Based on functional annotations and PheWAS, both genes with CpG sites may offer insights into the activation of genetic markers in diabetic CKD, suggesting that cg10297223 and cg02990553 could be considered as potential clinical biomarkers. Nevertheless, further studies are necessary to validate the association between whole blood and kidney tissue-specific DNA methylation.

## Supplementary Information

Supplementary Figures.

Supplementary Table 1.

Supplementary Table 2.

Supplementary Table 3.

Supplementary Table 4.

Supplementary Table 5.

## Data Availability

All the methylation array data have been deposited in the National Center for Biotechnology Information Gene Expression Omnibus under accession number GSE230652. The datasets of KNOW-CKD cohort are available from the corresponding author upon reasonable request. Summary statistics estimated in this study are available from Supplementary Table 2.
