# Supplementary material for: Epigenome-wide association study of diabetic chronic kidney disease progression in the Korean population: the KNOW-CKD study
Source: Sci Rep. 2023 May 20;13:8175. doi: 10.1038/s41598-023-35485-x (PMC10199928; doi:10.1038/s41598-023-35485-x)
Supplement: Supplementary file 1 — Supplementary Figures. [file 41598_2023_35485_MOESM1_ESM.docx]

**Supplementary Material Table of Contents**

Supplementary Figure 1. Flow of the study participants in this study. KNOW-CKD, The Korean Cohort Study for Outcomes in Patients with Chronic Kidney Disease; SNUH, Seoul National University Hospital; GN, glomerulonephritis; DN, diabetic nephropathy; HTN, hypertensive nephropathy; PKD, polycystic kidney disease; QC, quality control.

Supplementary Figure 2. Power calculation for the EWAS. The parameters as follows: 1:1 ratio of progression to non-progression, 780,000 total CpG sites, 800 targeted CpG sites, minimum detection of |∆ M-value| = 0.01, limma method, FDR threshold of 0.05, and 100 simulations. EWAS, epigenome-wide association study; CpG, Cytosine-phosphate-Guanine; FDR, false positive rate.

Supplementary Figure 3. Workflow of epigenome-wide association study for diabetic CKD progression. CKD, chronic kidney disease; CGI, CpG island; PheWAS, phenome-wide association study; BMI, body mass index.

Supplementary Figure 4. Circos plot of the epigenome-wide association study for diabetic CKD progression with epigenome-wide significance (FDR <0.05). (A) is not adjusted for blood cell proportions, (B) is adjusted for blood cell proportions, and (c) is adjusted for blood cell proportions, BMI, and smoking status. Red color is presented as hyper-methylated, while blue color hypo-methylated CpG sites, respectively. Outer bands are presented as neighboring distance. Two inner bands are presented as genomic density.

Supplementary Figure 5. The distribution of |∆ M-value| (upper) and the quantile estimates of top five percentile |∆ M-value| distribution (lower) based on the 1,000 times bootstrapping resampling method. (A) is not adjusted for blood cell proportions, (B) is adjusted for blood cell proportions, and (c) is adjusted for blood cell proportions, BMI, and smoking status. BMI, body mass index

Supplementary Figure 6. Workflow of pyrosequencing and functional analysis for diabetic CKD progression. CKD, chronic kidney disease; CGI, CpG island; PheWAS, phenome-wide association study; DNMT, DNA methyltransferase; RT-qPCR, reverse-transcription quantitative polymerase chain reaction; RA, Reactome; PPI, protein-protein interaction; eQTM, Expression Quantitative Trait Methylation.


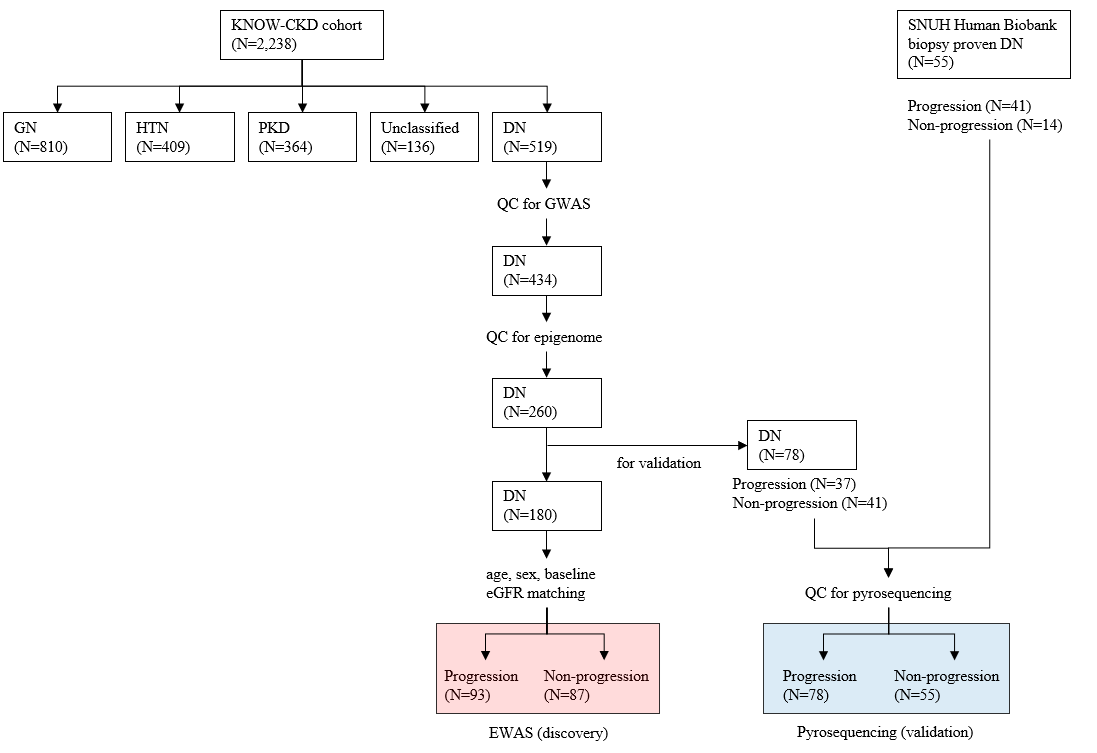


Supplementary Figure 1. Flow of the study participants in this study. KNOW-CKD, The Korean Cohort Study for Outcomes in Patients with Chronic Kidney Disease; SNUH, Seoul National University Hospital; GN, glomerulonephritis; DN, diabetic nephropathy; HTN, hypertensive nephropathy; PKD, polycystic kidney disease; QC, quality control.


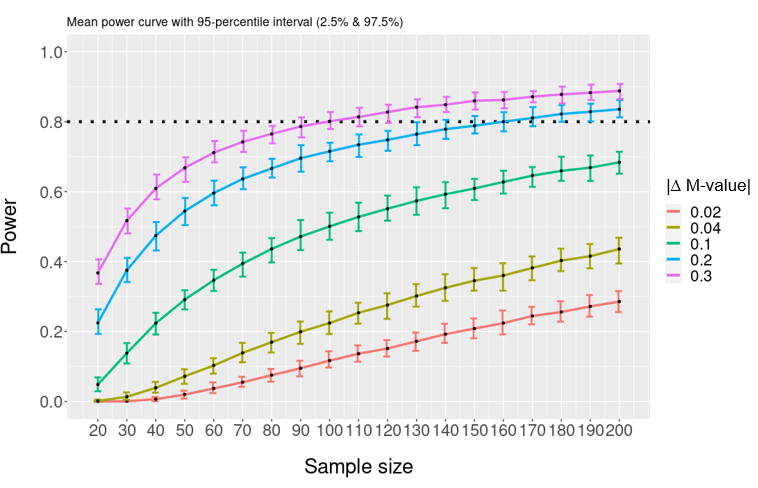


Supplementary Figure 2. Power calculation for the EWAS. The parameters as follows: 1:1 ratio of progression to non-progression, 780,000 total CpG sites, 800 targeted CpG sites, minimum detection of |∆ M-value| = 0.01, limma method, FDR threshold of 0.05, and 100 simulations. EWAS, epigenome-wide association study; CpG, Cytosine-phosphate-Guanine; FDR, false positive rate.


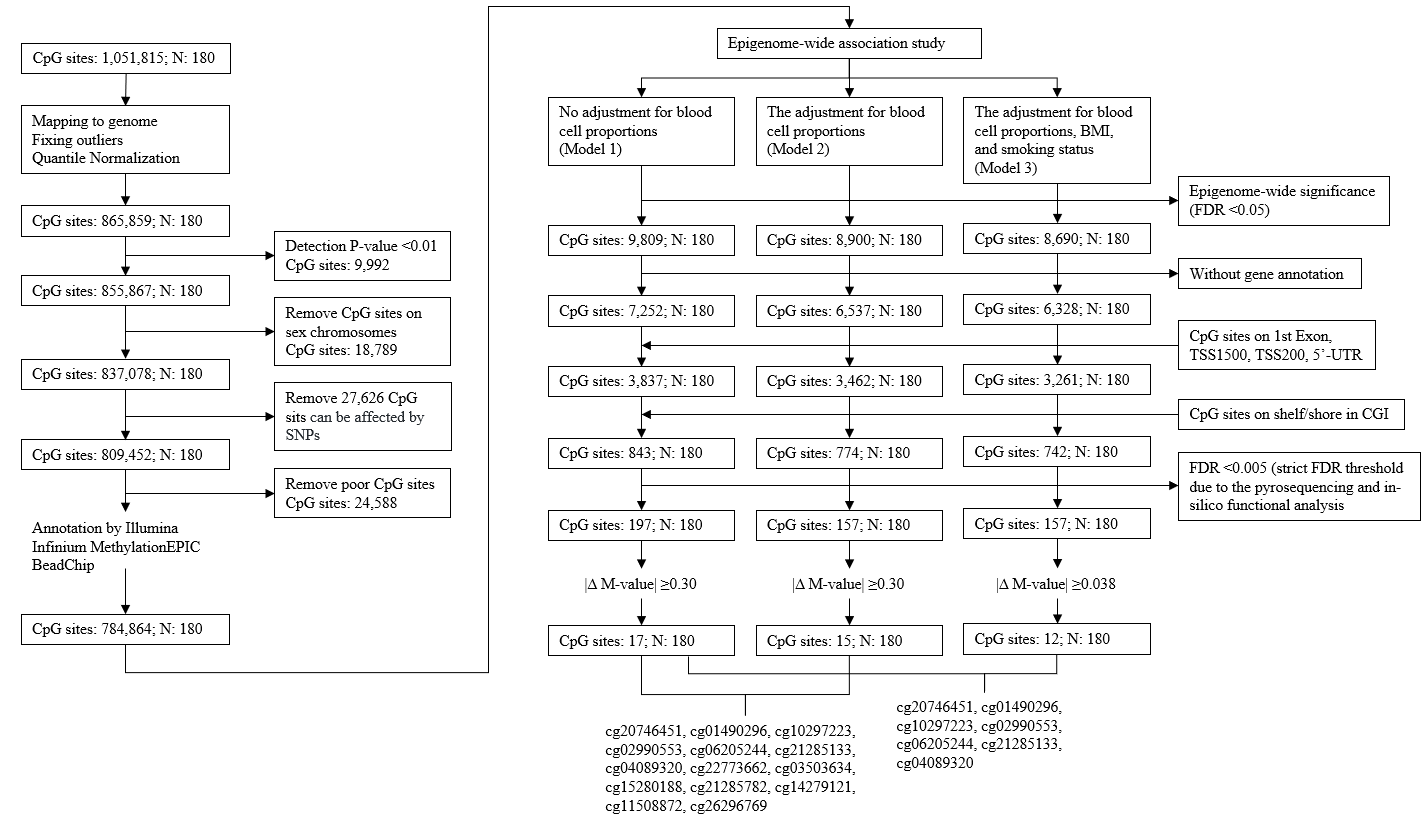


Supplementary Figure 3. Workflow of epigenome-wide association study for diabetic CKD progression. CKD, chronic kidney disease; CGI, CpG island; PheWAS, phenome-wide association study; BMI, body mass index.


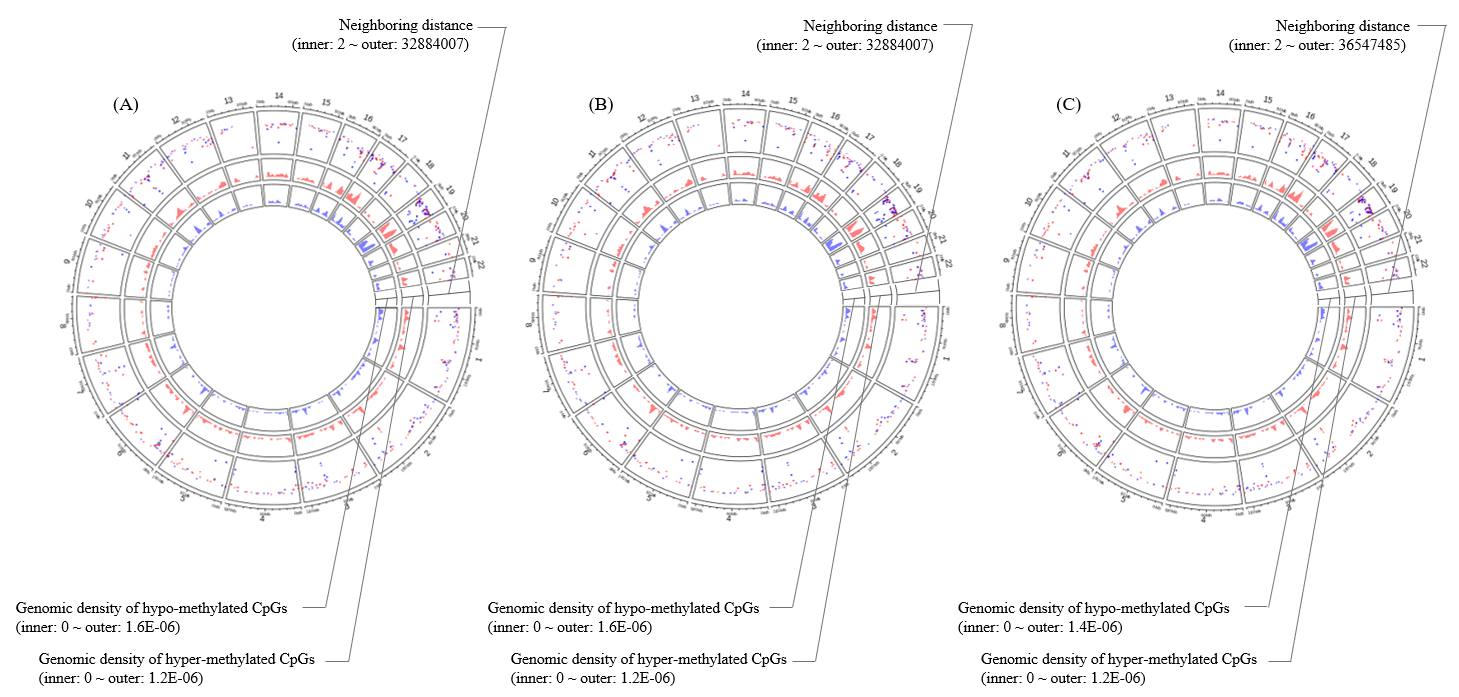


Supplementary Figure 4. Circos plot of the epigenome-wide association study for diabetic CKD progression with epigenome-wide significance (FDR <0.05). (A) is not adjusted for blood cell proportions, (B) is adjusted for blood cell proportions, and (c) is adjusted for blood cell proportions, BMI, and smoking status. Red color is presented as hyper-methylated, while blue color hypo-methylated CpG sites, respectively. Outer bands are presented as neighboring distance. Two inner bands are presented as genomic density.


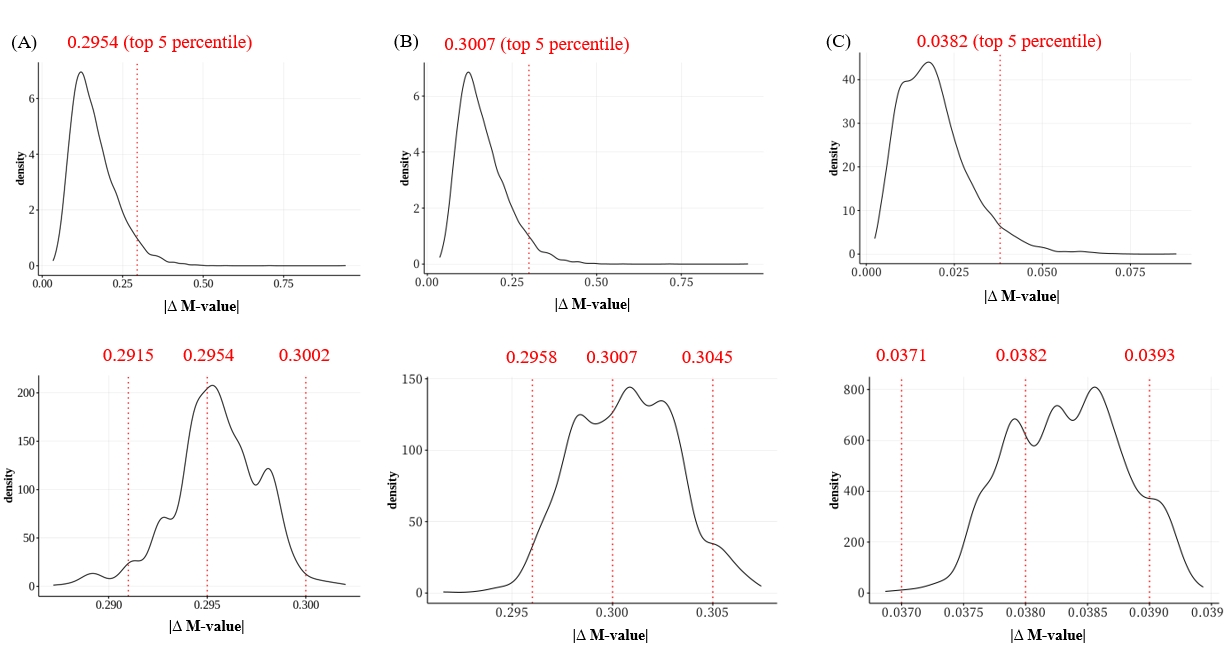


Supplementary Figure 5. The distribution of |∆ M-value| (upper) and the quantile estimates of top five percentile |∆ M-value| distribution (lower) based on the 1,000 times bootstrapping resampling method. (A) is not adjusted for blood cell proportions, (B) is adjusted for blood cell proportions, and (c) is adjusted for blood cell proportions, BMI, and smoking status. BMI, body mass index


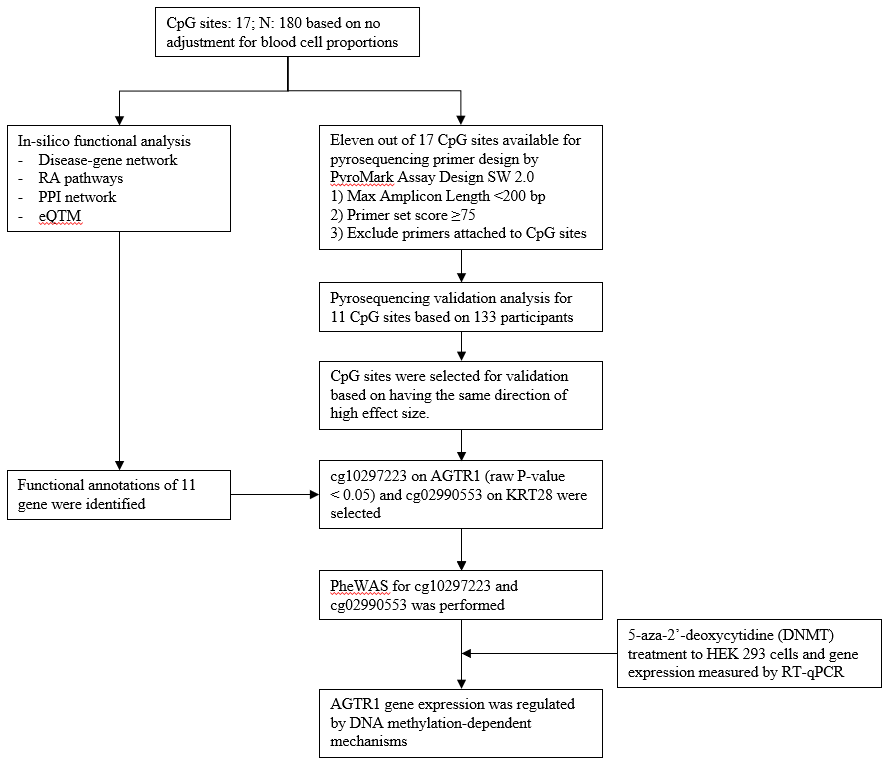


Supplementary Figure 6. Workflow of pyrosequencing and functional analysis for diabetic CKD progression. CKD, chronic kidney disease; CGI, CpG island; PheWAS, phenome-wide association study; DNMT, DNA methyltransferase; RT-qPCR, reverse-transcription quantitative polymerase chain reaction; RA, Reactome; PPI, protein-protein interaction; eQTM, Expression Quantitative Trait Methylation.
