# Supplementary material for: Epigenome-wide association study of diabetic chronic kidney disease progression in the Korean population: the KNOW-CKD study
Source: Sci Rep. 2023 May 20;13:8175. doi: 10.1038/s41598-023-35485-x (PMC10199928; doi:10.1038/s41598-023-35485-x)
Supplement: Supplementary file 2 — Supplementary Table 1. [file 41598_2023_35485_MOESM2_ESM.docx]

**Supplementary Table 1. General characteristics of diabetic chronic kidney disease based on the KNOW-CKD cohort study and the biopsy in the SNUH Human Biobank for pyrosequencing analysis.**

| **KNOW-CKD** |  |  |  |  |
| --- | --- | --- | --- | --- |
|  | Total  (N=78) | Progression  (N=37) | Non-progression  (N=41) |  |
|  | **Mean (SD)** | **Mean (SD)** | **Mean (SD)** | **P-value** |
| Age at baseline | 62.4 (13.7) | 61.1 (8.9) | 63.5 (8.7) | 0.22 |
| Systolic BP (mmHg) | 133.4 (18.2) | 137.2 (17.5) | 129.9 (18.3) | 0.07 |
| Diastolic BP (mmHg) | 74.9 (11.8) | 78.8 (10.8) | 71.5 (11.6) | <0.01 |
| Body mass index (kg/m^2^) | 25.1 (3.0) | 24.4 (2.9) | 25.6 (3.0) | 0.08 |
| White blood cells (/mm^3^) | 6876.0 (2036.3) | 7353.3 (2328.9) | 6456.8 (1657.4) | 0.06 |
| Hemoglobin (g/dL) | 12.3 (2.1) | 11.8 (1.9) | 12.7 (2.1) | 0.06 |
| Urine Albumin (mg/dL) | 1173.1 (1636.3) | 1782.3 (1688.6) | 610.8 (1382.6) | <0.001 |
| Urine Protein (mg/dL) | 163.6 (216.8) | 250.7 (229.5) | 83.2 (170.9) | <0.001 |
| UACR | 1.3 (1.6) | 2.1 (1.8) | 0.5 (1.0) | <0.001 |
| UPCR | 1.8 (2.3) | 2.9 (2.6) | 0.8 (1.3) | <0.001 |
| 24-h Urine protein (g) | 1.8 (2.7) | 3.0 (3.4) | 0.7 (1.1) | <0.001 |
| 24-h Urine Phosphorus (g) | 0.7 (0.6) | 0.7 (0.8) | 0.6 (0.2) | 0.20 |
| eGFR (ml/min/1.73m^2^) | 43.4 (18.0) | 43.3 (18.6) | 43.4 (17.7) | 0.97 |
| eGFR slope (ml/min/1.73 m^2^/year) | -2.4 (2.2) | -4.2 (1.5) | -0.8 (1.3) | <0.001 |
|  | **Median (IQR)** | **Median (IQR)** | **Median (IQR)** | **P-value** |
| Follow up (years) | 3.1 (1.8) | 2.9 (2.1) | 3.4 (1.5) | 0.30 |
|  |  |  |  |  |
|  | **N (%)** | **N (%)** | **N (%)** | **P-value** |
| Sex (male) | 57 (73.0) | 26 (70.3) | 31 (75.6) | 0.78 |
| Hypertension | 78 (100.0) | 37 (100.0) | 41 (100.0) | - |
| **SNUH Human Biobank** |  |  |  |  |
|  | Total  (N=55) | Progression  (N=41) | Non-progression  (N=14) |  |
|  | **Mean (SD)** | **Mean (SD)** | **Mean (SD)** | **P-value** |
| Age at baseline | 52.0 (13.7) | 52.3 (14.9) | 51.3 (11.0) | 0.78 |
| UACR | 3.3 (2.6) | 3.3 (2.1) | 3.2 (4.0) | 0.94 |
| UPCR | 5.6 (4.4) | 5.7 (3.7) | 5.4 (5.8) | 0.86 |
| eGFR (ml/min/1.73m^2^) | 52.7 (28.3) | 50.8 (29.0) | 58.2 (26.4) | 0.39 |
| eGFR slope (ml/min/1.73 m^2^/year) | -4.0 (3.3) | -5.1 (3.1) | -0.9 (1.3) | <0.001 |
|  |  |  |  |  |
|  | **N (%)** | **N (%)** | **N (%)** | **P-value** |
| Sex (male)† | 41 (74.5) | 28 (68.3) | 13 (92.9) | 0.09 |
| Hypertension | 33 (60.0) | 26 (63.4) | 7 (50.0) | 0.57 |

*Abbreviations:* KNOW-CKD, KoreaN cohort study for Outcome in patients With Chronic Kidney Disease; SNUH, Seoul National University Hospital; BP, blood pressure; eGFR, estimated glomerular filtration rate; IQR, interquartile Rang
† Fisher’s exact test
