# Supplementary material for: Epigenome-wide association study of diabetic chronic kidney disease progression in the Korean population: the KNOW-CKD study
Source: Sci Rep. 2023 May 20;13:8175. doi: 10.1038/s41598-023-35485-x (PMC10199928; doi:10.1038/s41598-023-35485-x)
Supplement: Supplementary file 4 — Supplementary Table 3. [file 41598_2023_35485_MOESM4_ESM.docx]

**Supplementary Table 3. The pyrosequencing analysis for the progression of diabetic chronic kidney disease**

| Gene | Primer (5’- 3’) | | Beta (SE) | P-value | FDR | Bonferroni  correction |
| --- | --- | --- | --- | --- | --- | --- |
| *DOC2A* | Forward | TGTTGTTGAGTTTGGGAAGAT | -0.091 (0.423) | 8.20E-01 | 0.85 | 1.00 |
|  | Biotinylated-reverse | AAATATAACTCTCCTACCAAATAAATTACC |  |  |  |  |
|  | Sequencing primer | TGAGTTTGGGAAGATATTAT |  |  |  |  |
| *AGTR1* | Forward | GTTGTTTGGGGTTTTGGTT | 0.788 (0.397) | 4.90E-02 | 0.59 | 0.59 |
|  | Biotinylated-reverse | CATATAAACTTTATCCATTTTTTACTTCT |  |  |  |  |
|  | Sequencing primer | TGTTAGGAGTTGGAAT |  |  |  |  |
| *MIEF1* | Forward | GGGTAGTTGATAGAATGAGTTGTTATGTGA | -0.076 (0.291) | 7.94E-01 | 0.85 | 1.00 |
|  | Biotinylated-reverse | AATACTTTATTACCCATAACCACATT |  |  |  |  |
|  | Sequencing primer | ATATGTTTAGAATATTGT |  |  |  |  |
| *TRAF6* | Forward | GGTGTAAAGAATTTTTAGGTGGGATAT | -0.054 (0.100) | 5.90E-01 | 0.85 | 1.00 |
|  | Biotinylated-reverse | ACCCCCTAACCATAATCCTCCAA |  |  |  |  |
|  | Sequencing primer | AATTTTTAGGTGGGATAT |  |  |  |  |
| *EMB* | Forward | GGTGGTTTTTTAGAAGTTGTTAGG | 0.330 (0.303) | 2.79E-01 | 0.84 | 1.00 |
|  | Biotinylated-reverse | CCAATCACACACATTCTCTCTTCTAATC |  |  |  |  |
|  | Sequencing primer | ATCCCAAAACCCCATA |  |  |  |  |
| *SMARCAD1* | Forward | TAGAGGTTGGGAAGGGTAGTG | 0.096 (0.472) | 8.40E-01 | 0.85 | 1.00 |
|  | Biotinylated-reverse | CCCTAAACAACAAAACAAAACTCTT |  |  |  |  |
|  | Sequencing primer | TGAGTATGGTTAATGGG |  |  |  |  |
| *OSBPL9* | Forward | TGGGGGTTGATGGAGTGA | -0.222 (0.133) | 9.84E-02 | 0.59 | 1.00 |
|  | Biotinylated-reverse | ACAAACCACCTACATTCCTTAT |  |  |  |  |
|  | Sequencing primer | GGGAGGAGGAGATATTA |  |  |  |  |
| *ASPSCR1* | Forward | GGGAAGTTTTTGAGTGTTTGTG | 0.101 (0.073) | 1.68E-01 | 0.67 | 1.00 |
|  | Biotinylated-reverse | CCCTCAATCTAAACCTAATTTCTTTC |  |  |  |  |
|  | Sequencing primer | TGGGTAGGTAGGAGTTT |  |  |  |  |
| *RAB14* | Forward | GTTTTTTTATGGATATAATGGGGATG | 0.033 (0.108) | 7.64E-01 | 0.85 | 1.00 |
|  | Biotinylated-reverse | AATTTTTAAAACCAACATTTACCACTT |  |  |  |  |
|  | Sequencing primer | ACCAACATTTACCACTTAT |  |  |  |  |
| *ANP32E* | Forward | TTAAATGGATTTGGGGAGTAGGAAG | -0.320 (0.540) | 5.50E-01 | 0.85 | 1.00 |
|  | Biotinylated-reverse | CCCCTAAAAAAATACACTATTCCTAA |  |  |  |  |
|  | Sequencing primer | ATAGGAGTTTGTTTGTTTAT |  |  |  |  |
| *KRT28* | Forward | AGAGAGAAAAATTAAGGGTTGGTATG | 0.459 (0.912) | 6.20E-01 | 0.85 | 1.00 |
|  | Biotinylated-reverse | TCTCACCTTATTCTTAAAATCCTCAATTAT |  |  |  |  |
|  | Sequencing primer | AAGGGTTGGTATGAAAA |  |  |  |  |
