# Supplementary material for: Epigenome-wide association study of diabetic chronic kidney disease progression in the Korean population: the KNOW-CKD study
Source: Sci Rep. 2023 May 20;13:8175. doi: 10.1038/s41598-023-35485-x (PMC10199928; doi:10.1038/s41598-023-35485-x)
Supplement: Supplementary file 6 — Supplementary Table 5. [file 41598_2023_35485_MOESM6_ESM.docx]

**Supplementary Table 5. The results of eQTM analysis based on the database of susztaklab (available on http://www.susztaklab.com/Kidney_meQTL/eQTM.php)**

| CpG | Gene symbol | Dis.CpG2TSS^1^ | Beta^2^ | P-value | FDR^3^ |
| --- | --- | --- | --- | --- | --- |
| CpG probes were validated | | | | | |
| cg15280188 | GPS1 | 73713 | 0.0767 | 0.00158 | 0.0393 |
| cg15280188 | PYCR1 | 34565 | 0.1176 | 0.00167 | 0.0393 |
| cg14279121 | TTC39A | 384109 | -0.118 | 0.00159 | 0.0159 |
| cg04089320 | PARP8 | 224092 | -0.05 | 0.000488 | 0.000977 |
| cg11513352 | PPP4C | 64762 | -0.0811 | 6.23E-7 | 2.68E-5 |
| cg11513352 | DOC2A | 12055 | -0.1507 | 1.44E-5 | 0.000309 |
| cg11513352 | TBX6 | 80709 | 0.1288 | 0.00065 | 0.00931 |
| cg11513352 | HIRIP3 | 15141 | -0.075 | 0.00228 | 0.0245 |
| cg11513352 | MVP | 190822 | -0.0872 | 0.00417 | 0.0359 |

eQTM, expression quantitative trait methylation; FDR, false positive rate.
1. Absolute distance betwwen CpG site and transcriptiaonl start site (TSS) of target gene.
2. eQTM Beta is reported as the association between CpG methylation and gene expression.
3. FDR is calculated based all tested pairs for each CpG
